# Association of Probiotics with Atopic Dermatitis among Infant: A Meta-analysis of Randomized Controlled Trials

**DOI:** 10.1155/2022/5080190

**Published:** 2022-05-23

**Authors:** Hua Pan, Jingqiu Su

**Affiliations:** Department of Dermatology, The Fourth Affiliated Hospital of China Medical University, 110004, China

## Abstract

**Background:**

Previous studies have explored the relationship between probiotics and risk of atopic dermatitis among infant; however, the results are still inconclusive. We aimed to assess the abovementioned association.

**Methods:**

PubMed, Web of Science, Embase, and China National Knowledge Infrastructure were retrieved for association between probiotics and atopic dermatitis with randomized controlled trials (RCTs) until Nov 20, 2021. The effect size was pooled by using random or fixed effect models according to the heterogeneity. Stata 12.0 was used for meta-analysis, sensitivity analysis, and bias analysis.

**Results:**

At the end of the screening article, 2575 infants were extracted from 8 trials and finally met the qualification criteria. In comparison to placebo, probiotics dramatically reduced incidence of childhood atopic dermatitis (RR = 0.86, 95% CI = 0.78-0.95). However, probiotics did not exhibit benefit over placebo in preventing the development of either IgE-associated infant AD (RR = 0.98, 95% CI = 0.79-1.22) or sensitive constitution (RR = 0.93, 95% CI = 0.81-1.08). From the results of sensitivity and publication bias, we found that these results were robust with little publication bias.

**Conclusion:**

During the late stages of pregnancy, women taking probiotics could lower the risk of infantile atopic dermatitis, but not for IgE-associated infant AD or sensitive constitution. The results could provide evidence for the fibrosis. Future studies are needed to confirm the results.

## 1. Introduction

Atopic dermatitis (AD) is one of the most common skin diseases in children. It could be divided into different stages: infant period, childhood period, and youth adult period. About 60% of patients with AD develop before the age of 1, and 85% develop AD before the age of five [1]. Nearly a quarter of children with the disease can be delayed into young adulthood [2]. In the past few decades, the incidence of the disease in the worldwide has increased significantly and is now 10-20 percent among infants and children [3]. Mancini et al. proposed in 2013 that the direct national expenditure on AD treatment in the United States was as high as 3.8 billion dollars, which costs $167 to $580 per person per year [4].

AD is related to genetic allergic diathesis, often accompanied by allergic rhinitis and asthma, and the three diseases are allergic progression, while atopic dermatitis is often the first symptom [5]. AD patients also have the following characteristics: easy to xenoprotein allergy, serum immunoglobulin-E (IgE) increased, and blood eosinophils increased [6]. The main treatment currently involves topical sugars corticosteroids, antihistamines, and even antibiotics, while long-term use of drugs can lead to side effects [7]. In addition, AD symptoms may recur rapidly after the stop of treatment. Therefore, prevention methods are needed in the face of recurrent AD. In addition, the association between AD and fibrosis has been studied for several years; however, the results between studies are controversial.

Previous studies revealed that gastrointestinal flora plays an important role in infant atopic dermatitis [8]. It is showed that the contents of bifidobacteria and lactobacillus decreased in the intestinal tract of AD patients, while the contents of clostridium increased [9]. All these evidences have indicated that probiotics may have a positive effect on the prevention and treatment of AD.

Since some researches have explored the effect of probiotics on AD, but there is little comprehensive analysis for the topic. Therefore, we conducted this research to overall analyze the effect of probiotics on AD.

## 2. Methods

### 2.1. Literature Search Strategy

We searched the randomized controlled trials from PubMed, Web of Science, Embase, and China National Knowledge Infrastructure up to Nov 20, 2021, using the following keywords: (1) probiotics, (2) atopic dermatitis, and (3) clinical effect. The search strategy involves medical subject headings (mesh) and text words combined by the Boolean operator “and.”

We will conduct a comprehensive search in multiple databases, regardless of the language or publication status. In order to maximize the specificity and sensitivity of the search, the author should also refer to the list of retrieved references to find other relevant studies not found through the search strategy. The literature search was conducted in accordance with PRISMA.

### 2.2. Study Selection

We conducted a comprehensive review of potentially relevant articles to ensure that they met all inclusion criteria: (1) study design was double-blind, randomized, placebo-controlled trials; (2) the intervention was probiotics or placebo; (3) mother during gestation and/or one year after birth received probiotics or placebo; (4) the outcome was AD or IgE related AD or sensitive constitution; (5) the full text is available for reference.

Studies were excluded according to the following predetermined exclusion criteria: (1) studies on other subjects; (2) comparison of other interventions; (3) lack of research on available data; (4) the outcome was the treatment effect, not the prevention effect; and (5) comments, abstracts, and reproduction of publications.

For studies with duplicate data from a single database, we selected the study with the most complete information on results or the largest sample size.

### 2.3. Data Collection and Quality Assessment

Two reviewers extracted detailed information including the titles, abstracts, and full-text articles of potentially qualified studies and resolved their differences through discussion. The following data parameters were extracted: name of main author, publication year, number of participants in each group, characteristics of drug intervention during follow-up in each group, and infants' age in the follow-up time. The Jadad questionnaire was used to evaluate the effectiveness of qualified randomized controlled trials. Egger's test and funnel plot program were used to assess the risk of bias in the study.

### 2.4. Statistical Analysis

The effect size was pooled by using random or fixed effect models according to the heterogeneity. Heterogeneity of effect size across studies was tested by *I*^2^ statistics (*I*^2^ > 50% is considered significant). The random effect model was used for analysis if *P* < 0.05 or *I*^2^ > 50%, or else fixed effect model was used to conduct the analysis. Visual examination of the funnel plot was used to assess publication bias. Sensitivity analysis was explored by deleting one study in turn to observe the impact of individual results on the overall analysis. The study was conducted by using STATA version 12.0. All *P* values are two tailed, and we set *P* < 0.05 as the threshold for significance.

## 3. Results

### 3.1. Search Process

**Figure 1:**
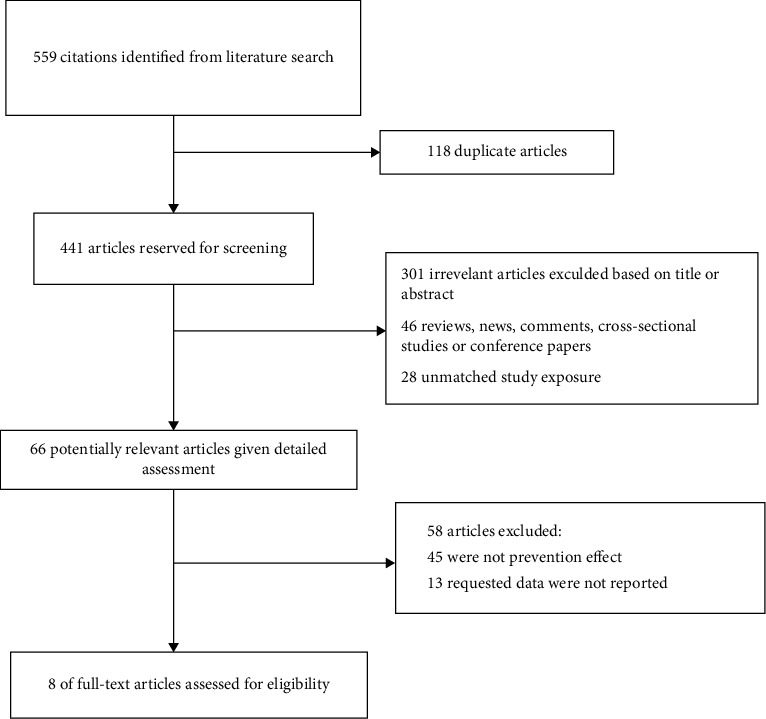
Flow chart for selection of eligible studies.

The initial search yielded 559 articles from four databases, including PubMed, Embase, Web of Science, and CNKI. After the first screening, 441 records were retained. By screening titles and abstracts, an additional 375 records were excluded because they were review articles, letters, case reports, comments, or editorials, remaining 66 articles. A total of 58 articles were further excluded for various reasons, including different research designs or insufficient available data. Finally, 8 studies met the inclusion criteria and were included in this meta-analysis [10–17], with a total of 2575 infants. The process followed PRISMA guidelines, including the reasons for excluding the study, as shown in Figure 1.

### 3.2. Characteristics of Included Studies

Table 1 lists the main characteristics of the eight studies. These studies included 2575 patients (1358 patients in the experimental group and 1217 patients in the control group). The sample size ranged from 115 to 891.

### 3.3. Results of Meta-analysis

#### 3.3.1. Association between Probiotics and AD

**Figure 2:**
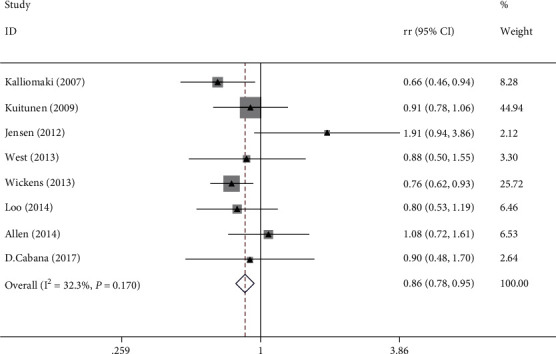
Association between probiotics and AD.

All the eight studies revealed the association between probiotics and AD; the overall results showed that the incidence of AD in the experimental group was lower than that of the control group (RR = 0.86, 95% confidence interval (0.78, 0.95), *P* = 0.170, *I*^2^ = 32.3% fixed effect model) (Figure 2).

#### 3.3.2. Association between Probiotics and Related AD

**Figure 3:**
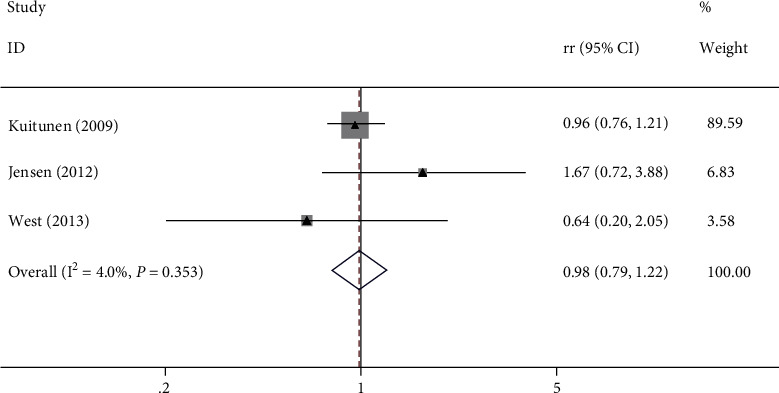
Association between probiotics and related AD.

Three studies revealed the association between probiotics and related AD; the overall results showed that the difference of incidence of related AD in the experimental group was not statistically significant from that of the control group (RR = 0.98, 95% CI (0.79, 1.22), *P* = 0.353, *I*^2^ = 4% fixed effect model) (Figure 3).

#### 3.3.3. Association between Probiotics and Sensitive Constitution

**Figure 4:**
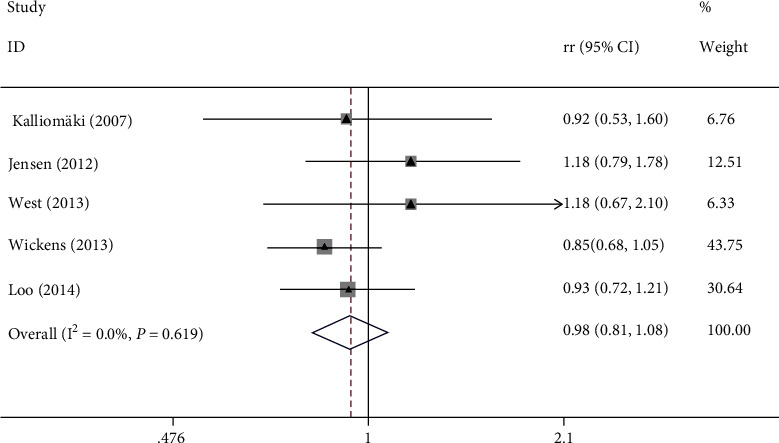
Association between probiotics and sensitive constitution.

Five studies revealed the association between probiotics and sensitive constitution; the overall results showed that the difference of incidence of sensitive constitution in the experimental group was not statistically significant from that of the control group (RR = 0.93, 95% CI (0.81, 1.08), *P* = 0.619, *I*^2^ = 0% fixed effect model) (Figure 4).

#### 3.3.4. Results of Quality Assessment

Jadad assessment tool was used to assess the quality in the study. All 6 literatures were analyzed from three aspects of “random grouping sequence generation method,” “allocation hiding,” and “blind method,” and all 8 articles were of high quality (Table 1).

### 3.4. Results of Sensitivity Analysis and Publication Bias

Sensitivity analysis was used to explore the potential sources of heterogeneity (Figure S1-S3). Excluding a single study in turn did not alter the combined RR significantly. Visual inspection of funnel plots did not identify substantial asymmetry (Figure S4-S6). No evidence of publication bias was found by Begg's and Egger's test.

## 4. Discussion

In this study, we found that the incidence of AD in the control group was higher than that of the probiotics group. However, the difference of incidence of related AD and sensitive constitution in the experimental group was not statistically significant from that of the control group. The results of our study may have important clinical practice, which indicated that probiotics may play a role in the prevention of AD, though it was not in the related AD and sensitive constitution.

AD is a chronic inflammatory skin condition associated with inherited allergic predisposition skin disease; the performance is pruritus, pleomorphic skin lesions, and exudate tendency, often concurrent asthma and allergic rhinitis. Studies have found that the allergic constitution of children in the intestines of E. coli number significantly more than the children allergic constitution, and allergic constitution of infants and young children, the intestinal microbial flora changes prior to the clinical manifestations of allergic disease speculated that the intestinal engraftment has proper number of bacteria and may be related to lower the risk of allergic diseases; improving the intestinal microecological environment in infants may help prevent allergic diseases in infants [18]. In addition, other results showed that after 1 year of age, children with eczema had more diversity of intestinal flora than normal children [19, 20]. The contents of bifidobacteria and lactobacillus were reduced in the intestines of AD patients. Pike et al. also found that intestinal permeability was increased in AD patients [21], which may be related to changes in intestinal flora. And the elevation of intestinal permeability may be related to the occurrence of food allergy. These theoretical evidences may suggest that exogenous supplementation of intestinal probiotics may be beneficial to the prevention and treatment of AD by changing the composition of intestinal flora.

The prevention mechanisms of probiotics on AD are still unknown. Probiotics can stimulate the production of intestinal IgA and reduce the adhesion of pathogenic bacteria and form a tight intercellular connection with intestinal epithelial cells to reduce intestinal permeability [22]. The toll-like receptor plays a role in inducing defensin, inhibiting the invasion of pathogenic bacteria and reducing local inflammation [23]. Exposure to specific skin pathogens can enhance the expression of thymic stromal lymphopoietin, which can induce the differentiation of primitive T cells into Th2 cells and Th17 cells, mediating allergic inflammation of the skin. Probiotics can act on DC cells and induce the production of regulatory T cells in mesenteric lymph nodes to migrate to inflammatory sites and produce IL-10 and TGF-*β*, thereby inhibiting Th2 and Th17 cell-mediated allergic reactions and the production of inflammatory factors and correcting the immune response to Th2 [24]. In our study, no significant difference was found in the incidence of IgE-related AD and the proportion of sensitive constitution between the probiotics group and the placebo group, suggesting that the probiotics' prevention effect on AD may not be mediated by IgE.

A few potential limitations of the present meta-analysis should also be acknowledged. First, baseline data are inconsistent in some literatures. Second, single language, different types, dosages, dosage forms, and study regions of probiotics may lead to biased results. Third, only eight studies were included in the meta-analysis, and the results should be further confirmed by more studies conducted in different populations.

## 5. Conclusion

In conclusion, our meta-analysis shows that probiotics may have a protective effect on the prevention of AD; the results of our study provide practical and valuable insights for the prevention of AD. Additional studies are needed to establish causality and elucidate the underlying mechanisms.

## Figures and Tables

**Table 1:** Characteristics of eligible studies.

Author	Year	Outcome	Number of population (trial/control)	Events (trial/control)	Types of probiotics	Intervention	Jadad score
Kalliomäki et al.	2007	AD, sensitive constitution	53/62	23/41	ATCC53103	Mother 2 ~ 4 weeks before prenatal start to take; postpartum use up to 6 months (not breast milk) feeders, to give to babies	6
Kuitunen et al.	2009	AD, related AD	445/446	175/193	ATCC53103 + DSM 7061 + DSM13692 + DSM7076	Starting two to four weeks before birth, babies are taken from birth until 6 months of age	7
Jensen et al.	2012	AD, related AD, sensitive constitution	62/56	19/9	LAVRI-A1	Babies are taken from birth until 6 months of age	7
West et al.	2013	AD, related AD, sensitive constitution	59/62	16/19	LF19	Babies take it from 4 months to 13 months	5
Wickens et al.	2013	AD, sensitive constitution	309/156	123/156	HN001 + HN019	Mothers take it from the 35th week until postpartum for 6 months; babies take it from birth to 2 years old	7
Loo et al.	2014	AD, sensitive constitution	124/121	31/38	BL999 + LPR	Babies are taken from birth until 6 months of age	5
Allen et al.	2014	AD	214/222	73/72	CUL61 + CUL08 + CUL34 + CUL20	Mothers take it from the 36th week until birth; babies take it from birth to 6 months	5
Cabana et al.	2017	AD	92/92	26/28	LPR	Babies are taken from birth until 6 months of age	5

## Data Availability

The datasets used and analyzed during the current study are available from the corresponding author upon reasonable request.

## Supplementary Materials

Supplementary Materials Figure S1: sensitivity analyses for studies of probiotics and AD. Figure S2: sensitivity analyses for studies of probiotics and related AD. Figure S3: sensitivity analyses for studies of probiotics and sensitive constitution. Figure S4: funnel plot for studies of probiotics and AD. Figure S5: funnel plot for studies of probiotics and related AD. Figure S6: funnel plot for studies of probiotics and sensitive constitution.
