# Supplementary material for: Association of Probiotics with Atopic Dermatitis among Infant: A Meta-analysis of Randomized Controlled Trials
Source: Oxid Med Cell Longev. 2022 May 23;2022:5080190. doi: 10.1155/2022/5080190 (PMC9150986; doi:10.1155/2022/5080190)
Supplement: Supplementary Materials — Figure S1: sensitivity analyses for studies of probiotics and AD. Figure S2: sensitivity analyses for studies of probiotics and related AD. Figure S3: sensitivity analyses for studies of probiotics and sensitive constitution. Figure S4: funnel plot for studies of probiotics and AD. Figure S5: funnel plot for studies of probiotics and related AD. Figure S6: funnel plot for studies of probiotics and sensitive constitution. [file 5080190.f1.docx]

**Supplementary material**

**Figure S1** Sensitivity analyses for studies of probiotics and AD

**Figure S2** Sensitivity analyses for studies of probiotics and related AD

**Figure S3** Sensitivity analyses for studies of probiotics and sensitive constitution

**Figure S4** Funnel plot for studies of probiotics and AD

**Figure S5** Funnel plot for studies of probiotics and related AD

**Figure S6** Funnel plot for studies of probiotics and sensitive constitution
